# Deficient neurotransmitter systems and synaptic function in frontotemporal lobar degeneration—Insights into disease mechanisms and current therapeutic approaches

**DOI:** 10.1038/s41380-021-01384-8

**Published:** 2021-11-19

**Authors:** Nadine Huber, Sonja Korhonen, Dorit Hoffmann, Stina Leskelä, Hannah Rostalski, Anne M. Remes, Paavo Honkakoski, Eino Solje, Annakaisa Haapasalo

**Affiliations:** 1 A.I. Virtanen Institute for Molecular Sciences, University of Eastern Finland, P.O. Box 1627, FI-70211 Kuopio, Finland; 2 Unit of Clinical Neuroscience, Neurology, University of Oulu, P. O. Box 8000, University of Oulu, FI-90014 Oulu, Finland; 3 MRC Oulu, Oulu University Hospital, P. O. Box 8000, University of Oulu, FI-90014 Oulu, Finland; 4 School of Pharmacy, University of Eastern Finland, P.O. Box 1627, FI-70210 Kuopio, Finland; 5 Department of Pharmacotherapy and Experimental Therapeutics, Eshelman School of Pharmacy, University of North Carolina at Chapel Hill, Chapel Hill, NC 27599 USA; 6 Institute of Clinical Medicine—Neurology, University of Eastern Finland, P.O. Box 1627, FI-70210 Kuopio, Finland; 7 Neuro Center, Neurology, Kuopio University Hospital, P.O. Box 100, KYS, FI-70029 Kuopio, Finland

**Keywords:** Diseases, Neuroscience

## Abstract

Frontotemporal lobar degeneration (FTLD) comprises a heterogenous group of fatal neurodegenerative diseases and, to date, no validated diagnostic or prognostic biomarkers or effective disease-modifying therapies exist for the different clinical or genetic subtypes of FTLD. Current treatment strategies rely on the off-label use of medications for symptomatic treatment. Changes in several neurotransmitter systems including the glutamatergic, GABAergic, dopaminergic, and serotonergic systems have been reported in FTLD spectrum disease patients. Many FTLD-related clinical and neuropsychiatric symptoms such as aggressive and compulsive behaviour, agitation, as well as altered eating habits and hyperorality can be explained by disturbances in these neurotransmitter systems, suggesting that their targeting might possibly offer new therapeutic options for treating patients with FTLD. This review summarizes the present knowledge on neurotransmitter system deficits and synaptic dysfunction in model systems and patients harbouring the most common genetic causes of FTLD, the hexanucleotide repeat expansion in *C9orf72* and mutations in the granulin (*GRN)* and microtubule-associated protein tau *(MAPT)* genes. We also describe the current pharmacological treatment options for FLTD that target different neurotransmitter systems.

## Introduction

Frontotemporal lobar degeneration (FTLD) comprises a heterogenous group of neurodegenerative syndromes characterized by progressive atrophy in the frontal and temporal lobes. Neurodegeneration in these brain areas is clinically associated with deficits in behaviour, progressive personality changes, executive dysfunction and/or understanding or producing speech. The clinical subtypes of FTLD include behavioural variant FTD (bvFTD) [1], nonfluent variant of primary progressive aphasia (nfvPPA), semantic variant of PPA (svPPA), logopenic variant of PPA (lvPPA) [2] as well as progressive supranuclear palsy (PSP) and corticobasal syndrome (CBS) [3]. FTLD is commonly associated with parkinsonism [4] and a great number of FTLD patients also present neuropsychiatric symptoms [5] or motor neuron dysfunction meeting the criteria of amyotrophic lateral sclerosis (ALS) [6, 7]. Several genetic mutations are associated with FTLD such as those in the granulin (*GRN)* and microtubule-associated protein tau *(MAPT)* genes [2]. However, the major genetic cause underlying both FTLD and ALS is a GGGGCC_(n)_ hexanucleotide repeat expansion in the *C9orf72* gene (C9-HRE) [8–10]. Unlike in the other types of FTLD, genetics do not play a major role in CBS or PSP [11].

Currently, no specific validated diagnostic or prognostic biomarkers or effective disease-modifying therapies are available for FTLD which complicates the correct and timely diagnosis and treatment. However, FTLD is accompanied by changes in several neurotransmitter systems including the glutamatergic, GABAergic, dopaminergic, and serotonergic systems [12] which are linked with specific neuropsychiatric symptoms such as aggressive and compulsive behaviour, agitation, and altered eating habits and hyperorality [12]. Due to the lack of officially approved pharmacological therapies for FTLD in Europe and the US, off-label medications targeting neurotransmitter systems to alleviate the clinical symptoms are commonly used. Nevertheless, these treatments do not target the pathogenic mechanisms of FLTD, and there is limited evidence for their ability to slow disease progression.

## Molecular mechanistic evidence from genetic model systems supporting synaptic dysfunction in FTLD

FTLD is suggested to have a strong genetic background and approximately 40% of patients have a family history with at least one affected family member [13]. Heritability varies between the different FTLD clinical subtypes. It is suggested that heritability plays a role in 48% of patients with bvFTD but only in 12% of svPPA patients [14]. Overall, the C9-HRE accounts for ~25% familial FTLD and ~6% of sporadic FTLD cases, although the frequency largely varies in geographically different populations [8]. Different *MAPT* mutations can be the underlying cause of up to 50% of familial and ~10% of sporadic FTLD patients and ~10% of FTLD cases are caused by loss of function mutations in *GRN*, but also the variant frequencies in these genes may differ between different geographical areas [14, 15]. Other genetic mutations have also been found to associate with FTLD, but the C9-HRE and mutations *GRN* and *MAPT* represent the three most common ones [14, 15].

### C9-HRE

The C9-HRE, identified in 2011, is a major cause of both FTLD and ALS [9, 10]. C9-HRE most commonly associates with bvFTD and bvFTD with ALS clinical phenotypes [9, 10, 16]. Suggested pathological mechanisms of the C9-HRE are *C9orf72* loss-of-function due to haploinsufficiency that leads to a decreased expression of the C9orf72 protein, and toxic gain-of-function caused by the accumulation of pathological RNA foci and dipeptide repeat-containing (DPR) proteins (poly-GA, poly-GP, poly-GR, poly-PA and poly-PR) [17–19].

Even though the C9orf72 protein is important in the regulation of key cellular and membrane trafficking events and actin cytoskeleton dynamics, *C9orf72* loss-of-function does not appear to be a primary disease-driving mechanism in FTLD or ALS [20]. The *C9orf72* knockout mouse models do not exhibit neurodegeneration, but display immune system dysfunction and develop autoimmune disease-like phenotypes, suggesting that *C9orf72* is a central regulator in the immune system [21–23]. However, loss of *C9orf72* has been shown to lead to the accumulation of DPR proteins through impaired lysosomal function and vesicle trafficking, which might cause neurotoxicity [24]. In addition, loss of *C9orf72* function leads to the accumulation of glutamate receptors and excitotoxicity [24], which alone or in combination with e.g., DPR proteins could underlie synaptic alterations.

Deficits in neurotransmission have been detected in the CNS and neuromuscular junctions of C9-HRE carriers [25]. Alterations in neuronal excitability, morphology, and excitotoxicity have also been reported in different C9-HRE model systems [26]. Regulation of RNA metabolism is crucial for local synaptic protein synthesis and synaptic function, and dysregulation of this system leads to neuronal hyperexcitability [27]. Interestingly, the GGGGCC_(n)_ repeat-containing RNA was actively transported into neurites, which resulted in decreased number of dendritic branches in *Drosophila* or rat spinal cord neurons [28]. In addition, overexpression of the GGGGCC_(n)_ repeat expansion caused synaptic and neuromuscular junction deficits [29]. In line with these findings, overexpression of the poly-GA DPR protein in primary mouse cortical neurons led to deficits in dendritic spine formation [30], reduced synaptic vesicle-associated protein 2 (SV2) levels, and altered Ca^2+^ influx and release of synaptic vesicles in mice [31]. Similar defects have been detected in other C9-HRE models and models having alterations in certain RNA-binding protein species [32, 33].

Excitotoxicity is associated with synaptic dysfunction in ALS and probably also in FTLD [26]. The excitotoxic mechanisms include dysfunction of the α-amino-3-hydroxy-5-methyl-4-isoxazolepropionic acid (AMPA) or N-methyl-D-aspartate (NMDA) glutamate receptors, leading to excess Ca^2+^ influx and cell death. The research on excitotoxicity has mainly focused on AMPA receptors, which regulate fast synaptic transmission and synaptic plasticity [34]. The AMPA receptor consists of four subunits termed GluA1-4. GluA2 is responsible for regulating Ca^2+^ permeability and AMPA receptor trafficking. Usually, GluA2-containing AMPA receptors are impermeable to Ca^2+^, but insufficient editing of GluA2 RNA can lead to increased Ca^2+^ permeability and cell death [35]. Double-stranded RNA-specific editase B2 (ADARB2) modifies the GluA2 subunit, and its absence leads to neuronal death. In line with this, ADARB2 has been shown to interact with the GGGGCC_(n)_-containing RNA and sequester within the RNA foci in C9-HRE-carrying iPSC-derived neurons that are susceptible to glutamate-mediated excitotoxicity [36]. Contribution of the NMDA receptors in C9-HRE FTLD and ALS is understudied but findings in different genetic ALS models suggest a role for NMDA receptors in neurodegeneration [26]. Also, blockade of NMDA receptors in presynaptic glutamatergic motor neurons rescued the motor deficits and the short life span caused by poly-GR/PR expression in a *Drosophila* model [37]. Furthermore, C9-HRE-carrying iPSC-derived motor neurons display upregulated levels of Ca^2+^-permeable AMPA and NMDA receptor subunits and significantly increased Ca^2+^ transients upon depolarization, repeated firing in response to glutamate, and sustained high concentrations of cytosolic Ca^2+^ in association with increased susceptibility to cell death [32].

Neuronal hyperexcitability and subsequent hypoexcitability during disease progression is characteristic for ALS [26]. The hyperexcitability can be caused by altered GABAergic signalling in inhibitory neurons together with alterations of Na^+^ and K^+^ channel functions and extracellular K^+^ levels [33]. Human iPSC-derived neurons carrying the C9-HRE show initial hyperexcitability followed by declined synaptic function [29]. Furthermore, overexpression of poly-GR led to the suppression of neuronal excitability [26]. The hyper- and hypoexcitability in ALS and FTLD patients vary greatly in different neurons (cortical *vs*. motor neurons) and disease states (presymptomatic *vs*. active disease). Finally, supporting synaptic dysfunction at the neuronal network level, which could contribute to the neurodegenerative processes in patients, a recent study showed significant alterations in the network activity of iPSC-derived cortical neurons from C9-HRE carriers. This included enhanced network burst activity due to increased synaptic contacts and impaired presynaptic synaptic vesicle dynamics and synaptic plasticity [38]. All in all, these studies in different model systems collectively suggest that the C9-HRE is associated with alterations at synapses at the molecular, functional, and neuronal network activity levels.

### GRN

Another major underlying cause of FTLD are the *GRN* loss-of-function mutations and the resulting haploinsufficiency leading to decreased levels of progranulin proteins. FTLD associated with *GRN* mutations is clinically heterogenous and neuropathologically characterized by ubiquitin and TDP-43-immunoreactive cytoplasmic inclusions [39]. Of the clinical phenotypes, bvFTD most commonly associates with *GRN* mutations and the patients usually present a combination of behavioural abnormality and language disturbance that is most often nfvPPA or PPA with a mixed phenotype [14]. The progranulin protein is involved in development, wound repair, and modulation of inflammation. In the CNS, progranulin expression is the highest in the cerebral cortex, hippocampus, and cerebellum, suggesting that reduced levels could affect neuronal survival as well as CNS inflammatory processes [40]. The *GRN* mutations have also been shown to alter synaptic function. *Grn* knockout mice show altered synaptic connectivity and impaired synaptic plasticity. In addition, pyramidal neurons in the hippocampus of the *Grn* knockout mice display altered neuronal morphology and synaptic transmission as well as significantly decreased dendric spine density compared to wild-type mice [41]. *Grn* depletion leads to diminished NMDA receptor density and NMDA-dependent tau phosphorylation, both leading to reduced neuronal arborization [42]. Moreover, studies in *Grn-*deficient mice showed that lysosomal dysregulation leads to neuroinflammation, synaptic loss, and decreased number for markers of oligodendrocytes, myelin, and neurons. Interestingly, upregulation of the lysosomal transmembrane glycoprotein NMB (GPNMB) levels, observed in the *Grn*-deficient mice in the same study, was also specifically detected in the brain and cerebrospinal fluid of *GRN* mutation carriers, implicating that it could be a potential discriminative biomarker for the differentiation of *GRN* mutation-carrying FTLD patients from the *MAPT* mutation or C9-HRE carriers [43]. *Grn* knockout mice have also been reported to show profound microglia infiltration and elimination of inhibitory synapses, which leads to hyperexcitability [44]. Moreover, knockdown of *Grn* in primary rat hippocampal neurons resulted in reduced neuronal connectivity because of decreased neuronal branching and branch length as well as diminished synaptic density [45]. Concomitantly increased synaptophysin, VGAT, and vGlut-1 puncta, associated with an increase in the number of synaptic vesicles per synapse, suggested an increase in the probability of release at the remaining synapses. Similar findings have been made in *post-mortem* brain samples of FTLD patients with *GRN* haploinsufficiency, possibly reflecting a compensatory mechanism related to synaptic loss [45].

### MAPT

The first mutation in the *MAPT* gene, encoding the microtubule-associated tau protein, was identified in 1997 and linked to FTLD with parkinsonism [46]. Patients with *MAPT* mutations present atrophy in the frontal and temporal lobes and basal ganglia and are neuropathologically characterized by tau-positive inclusions, consisting of toxic intracellular aggregates of hyperphosphorylated tau, in the brain (FTLD‐tau). Alternative splicing of the *MAPT* gene generates six different tau protein isoforms. Pathological inclusions of tau containing either three (3 R) or four (4 R) microtubule-binding domains are characteristic in the brains of *MAPT* mutation carriers, depending on the clinical subtype [47]. *MAPT* mutation-carrying patients may show behavioural changes, semantic impairment, and memory decline accompanied by parkinsonism [48]. *MAPT* mutations most often associate with bvFTD but rarely with different types of PPA [14]. Tau expression is the highest in neurons compared to other cell types throughout the CNS. Healthy neurons maintain a spatial gradient of tau, whose concentration is greater in axons than in the somatodendritic compartment [49]. Inversion of this gradient leads to disturbed axonal transport [50]. In addition, changes in the tau gradient alters the actin cytoskeleton and the direct interaction between tau and actin is suggested to mediate tau-induced neurotoxicity [51]. A disturbed gradient also leads to the accumulation of tau in the dendritic spines and synapses, which causes synaptic dysfunction and degeneration of axons and neurons [52]. Expression of human mutant tau in mice revealed that early tau-related symptoms such as early memory deficits and disrupted synaptic plasticity develop due to the synaptic abnormalities caused by the missorting and accumulation of hyperphosphorylated tau within dendritic spines rather than due to the loss of synapses or neurons. Mutant tau expression also disrupts synaptic function by impairing AMPA and NMDA receptor trafficking and synaptic anchoring and AMPA receptor-mediated synaptic responses [53].

Tau pathology is a central pathological hallmark of Alzheimer’s disease (AD), the most common tauopathy. It has been suggested that tau can spread from cell to cell through neuronal connections in the AD brain, and this process is accelerated in the presence of β-amyloid (Aβ) in both animal models and humans [54]. This propagation of tau pathology throughout the brain using a prion-like mechanism is thought to involve several steps, including cellular uptake, seeding, secretion, and intercellular transfer through synaptic and non-synaptic pathways [55]. Interestingly, emerging evidence supports the theory that misfolded tau exhibits prion-like properties, including the ability to seed and self-propagate along the axons, also in the brains of patients with other tauopathies, such as FTLD [56], suggesting partially similar neurodegenerative processes between AD and FTLD.

## Neurotransmitter system deficits in FTLD patients

**Fig. 1:**
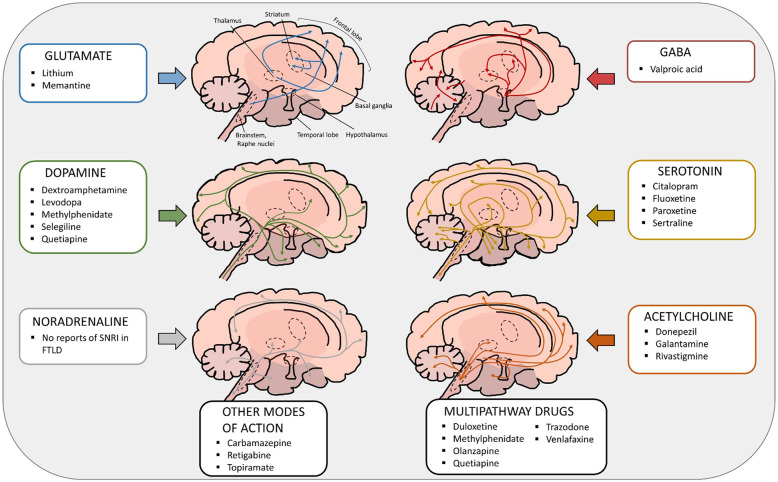
Neurotransmitter pathways involved in FTLD and current therapeutics affecting the different neurotransmitter systems. FTLD has been linked to changes in several principal neurotransmitter systems such as the glutamatergic, GABAergic, dopaminergic and serotonergic systems. Due to the lack of officially validated and approved pharmacological therapies for FTLD, off-label medications targeting different neurotransmitter systems, indicated here, to alleviate the clinical symptoms are commonly used. Drugs with other modes of action are also in use. For instance, retigabine acts as a positive allosteric modulator of the neuronal potassium channels KNCQ (Kv2 to 5), carbamazepine preferentially binds to voltage-gated sodium channels in their inactive conformation, which prevents repetitive and sustained firing of an action potential, and topiramate blocks voltage-dependent sodium and calcium channels. There are several drugs (listed in the multipathway drug box) that act simultaneously on different pathways. For example, methylphenidate and quetiapine affect both dopaminergic and noradrenergic pathways. Also, antidepressants such as duloxetine and venlafaxine affect the noradrenergic system but have also other targets. Some drugs have multiple targets such as trazodone (serotonin, histamine, and α1-adrenergic receptors, serotonin reuptake) and olanzapine (dopamine, serotonin, α1-adrenergic, muscarinic receptors). The mode of action of olanzapine is not completely clear and it may possibly act through the dopaminergic, serotonergic or cholinergic pathways. Currently there are no selective serotonin-noradrenaline re-uptake inhibitors (SNRI) targeting the noradrenaline system on the market.

Patients with FTLD spectrum diseases have been reported to display changes in several principal neurotransmitter systems such as the glutamatergic, GABAergic, dopaminergic and serotonergic systems [57]. The different neurotransmitter system pathways are depicted in Fig. 1. The effects of individual neurotransmitter system deficits in the CNS of patients with FTLD spectrum diseases are described in more detail in the following chapters and summarized in Table 1.

**Table 1:** Summary of neurotransmitter system deficits in FTLD.

Neurotransmitter pathway	Deficit	References
Dopamine		
Dopaminergic neurons	Reduced number of neurons in nigrostriatal pathway, low dopamine levels	[67]
Dopaminergic receptors	Reduced levels of D2 dopamine receptors	[12]
Serotonin		
Serotonergic neurons	Loss of neurons and tau deposition in raphe nucleus	[77]
Serotonergic receptors	Number of receptors reduced in midbrain, frontal and temporal lobes	[75]
Glutamate		
Glutamatergic neurons	Neurons lost in the thalamus, frontal and temporal cortex, reduced glutamate levels in frontal and temporal lobes	[60]
Glutamatergic receptors	AMPA and NMDA receptor levels decreased in frontal and temporal lobes, metabotropic glutamate receptors reduced in cerebral cortex, basal ganglia and thalamus	[56, 119]
GABA		
GABAergic neurons	Neurons reduced in frontal and temporal lobes	[57, 60]
GABAergic receptors	No evidence available of deficits in receptors	
Noradrenaline		
Noradrenergic neurons	No clear evidence for neuronal loss, minimal loss observed in locus coeruleus	[77]
Noradrenergic receptors	No evidence for chance in noradrenergic receptors	[76]
Acetylcholine		
Cholinergic neurons	Reduced cholinergic neurons in the nucleus basalis, no evidence of neuronal loss in cerebral cortex	[83]
Cholinergic receptors	Conflicting results	[130, 131]

### Glutamatergic system

Glutamate is the main excitatory neurotransmitter that acts through ionotropic glutamate receptors (NMDA, AMPA and kainate) and G protein-coupled metabotropic glutamate receptors [58]. Glutamate is crucial for memory formation, higher cognitive functions and regulation of NMDA receptor-mediated hippocampal long-term potentiation. Several NMDA receptor antagonists impair cognitive functions and may cause hallucinations. In contrast, excess glutamate may cause excitotoxic neuronal death and enhanced neurodegeneration. There are preclinical and clinical evidence for glutamatergic dysfunction in FTLD. Glutamate-mediated excitotoxicity is a possible disease-driving mechanism in AD and might also contribute to FTLD pathogenesis including that related to e.g., C9-HRE [59]. The number of glutamatergic neurons is decreased in the frontotemporal area and thalamus [60], and both ionotropic (NMDA and AMPA) and metabotropic glutamate receptors are reduced in FTLD spectrum patients. In addition, impairment of the glutamatergic neurotransmitter system is a typical finding in transcranial magnetic stimulation (TMS) measurements in FTLD patients [57, 61]. FTLD patients may display an abnormal composition of the AMPA receptor, as a reduction of GluA3 subunit-containing AMPA receptors has been detected in FTLD patients due to the formation of anti-GluA3 antibodies [62]. These patients often showed the clinical phenotype of bvFTD and an earlier onset of the disease. This is interesting because the lack of these AMPA receptor subtypes is known to result in increased dopamine levels in the striatum, reduced serotonin turnover in the olfactory bulb and aggressive behaviour in mice [63]. These results point to an association between AMPA receptors and the dopaminergic and serotonergic networks, which may be mechanistically important in FTLD pathogenesis.

### GABAergic system

Gamma-aminobutyric acid (GABA) is the main inhibitory neurotransmitter, which acts through two receptor subclasses: ionotropic (GABA_A_) and G protein-coupled GABAergic receptors (GABA_B_) [64]. GABAergic neurons are important in balancing neuronal circuit function. Thus, dysfunction in the GABAergic system might lead to poor information processing and altered behaviour, such as schizophrenia-like behaviour, motor dysfunction function, altered reproduction, aggressive-defensive behaviours and changes in working memory [65]. There is evidence for GABAergic neuron loss in the frontal and temporal cortex of FTLD patients [60], and the detection of impairment of the GABAergic system has been used in FTLD diagnostics using TMS [57, 61].

### Dopaminergic system

Dopamine is synthesized in dopaminergic neurons, which project throughout the brain. Dopamine acts through five known G protein-coupled receptors in the CNS and peripheral neurons [66]. Defects in the dopaminergic system are common in FTLD as there is an evident loss of presynaptic dopaminergic and nigrostriatal neurons [12]. The nigrostriatal pathway is important for movement and motor control and this pathway is degenerated for instance in Parkinson’s disease (PD). In line with this, extrapyramidal symptoms similar to PD are reported in up to 40 % in patients of FTLD spectrum on their first physician consultation. Parkinsonism is common among bvFTD patients, but it can be detected in all FTLD subtypes [67], and in general, up to 50 % of patients show parkinsonism symptoms [68, 69]. It has been postulated that the loss of mesocortical dopaminergic tracts and dopamine receptors in the frontal lobes could contribute to the behavioural symptoms in FTLD [12]. Moreover, dopamine release is involved in eating disorders and responsible for food reward [70]. Binge eating and increased alcohol consumption is a common clinical feature bvFTD, and therefore makes it distinguishable from other forms of dementia [71]. Many patients change their eating habits during disease progression, leading to compulsive overeating.

### Serotonergic system

Serotonin (5-HT) is synthesized in the raphe nuclei, which project widely to the brain and spinal cord [72]. It regulates several higher cognitive functions including feeding behaviour, metabolism and energy balance [73]. Seven different serotonin receptor families are known, but due to the subtype-specific genetic polymorphisms and complexity of the serotonergic networks, research in FTLD has mainly focused on 5-HT_1A_ and 5-HT_2A_ receptors [12]. Furthermore, serotonin is an important neuromodulator of other neurotransmitters, and it regulates synaptic plasticity. Clinical signs of serotonergic dysfunction may include behavioural disturbances, apathy, depression, and anxiety [74]. Indeed, reduced serotonergic activity is especially associated with several behavioural symptoms detected among bvFTD patients. For example*, post-mortem* studies have indicated a reduction of 5-HT_1A_ and 5-HT_2A_ receptors in the frontotemporal area and hypothalamus [75]. Furthermore, despite normal or elevated serotonin levels in the *post-mortem* analysis, serotonin turnover appears disturbed in bvFTD patients [76]. There is also evidence for the loss of ascending serotonergic pathways and a 40 % reduction of neurons in the raphe nuclei in FTLD [77]. These differences between reduced receptor densities and normal neurotransmitter levels might refer to a functional compensation for the loss of serotonergic neurons at different stages of the disease. Thus, dysfunction of the serotonergic system might contribute to the clinical symptoms of FTLD spectrum diseases by disturbing the neuromodulatory effects of the serotonin network and subsequently affecting other neurotransmitter systems.

### Noradrenergic system

Noradrenaline is synthesised in the locus coeruleus in the pons [78]. Noradrenergic pathways originating from locus coeruleus project especially to the forebrain and regulate, for instance, memory, attention, and decision-making but especially alertness and inactivity phase. Noradrenaline acts via G protein-coupled α- and β-adrenergic receptors. These receptors have excitatory or inhibitory effects depending on their synaptic location. For example, presynaptic α2-receptors can enhance the noradrenergic stimulus [79, 80]. Elevated noradrenaline levels decrease impulsivity and reduced noradrenergic transmission is associated with impaired executive function in rats [81]. Thus, due to intricacies of the noradrenaline signalling network, pharmacological interventions might result in complex behavioural outcomes. Despite the likely decreased metabolism and turnover of noradrenaline, there is no clear evidence for the loss of noradrenergic neurons or receptors in bvFTD patients [76]. However, the differences in research methods, brain regions investigated and the interplay among neurotransmitter systems might affect the findings. For example, the loss of serotonergic neurons might result in increased noradrenergic signalling [80].

### Cholinergic system

Acetylcholine is mainly synthesized in the nucleus basalis of Meynert and regulates several cognitive functions such as memory, emotion, speech production, attention and motor control [82]. The two main acetylcholine receptor classes, the ion channel-gated nicotinic receptors and the G protein-coupled muscarinic receptors, mediate excitatory or inhibitory inputs depending on the receptor type and its pre- or postsynaptic localisation. The cholinergic pathways do not play a major role in bvFTD [57, 61], but especially in PPA, varying results on the cholinergic receptor loss are reported [83, 84]. However, cholinesterase inhibitors do not improve cognitive function or alleviate other symptoms of FTLD patients [3], and may even reinforce behavioural symptoms [85]. However, the AD drug galantamine has been reported to alleviate language deficits in PPA patients [86].

## Functional neuronal network deficits in FTLD patients

Synaptic dysfunction may also be linked to deficits in neuronal network activity and dysconnectivity in core FTLD brain regions. However, such studies in FTLD are so far relatively rare. Intrinsic connectivity networks are temporally synchronous, low frequency fluctuations in the blood oxygen level-dependent signal, which correspond to spatial neural sets activated in task-activation functional magnetic resonance imaging (fMRI) studies [87, 88]. In various neurodegenerative diseases, functional intrinsic connectivity network deficits linked to clinical symptoms have been detected even in the absence of atrophy in the corresponding structural brain regions [89, 90]. Deficits in several intrinsic connectivity networks have also been indicated in FTLD spectrum diseases. Salience network, which has an essential role in the processing of consecutive responses to emotional and sensory stimuli, and which primarily adheres to the anterior insula, dorsal anterior cingulate cortex, amygdala, ventral striatum, and medial thalamus, has been shown to present deficits in different forms of FTLD [91–94]. A study by Lee and colleagues indicated that the behavioural symptoms in both C9-HRE-carrying and sporadic bvFTD patients correlated with deficits in the connectivity in the salience network [88]. Interestingly, the same study reported that sporadic FTLD patients had increased connectivity in the default mode network. This network is responsible for functions that are activated when an individual does not actively concentrate on the surrounding world. Default mode network alterations have also been observed in *GRN* mutation carriers who have a mood disorder [95]. Moreover, activity of the frontoparietal network, which is important in executive functions, appears to highly differ between *GRN* mutation-carrying FTLD-patients and healthy controls [96]. Finally, impairment of frontolimbic network, which is pivotal in emotion processing and moderating motivated behaviours, has been detected both in bvFTD and PPA patients and correlate with apathy [92]. Taken together, the observed deficits in the different neurotransmitter systems appear to be generally linked to wide dysfunction of the different functional networks in the brains of FTLD patients and underlie the altered behaviour or other clinical symptoms in these patients.

## Current pharmacological treatment strategies for patients suffering from FTLD

Currently, there are no officially approved medicinal therapies for FTLD spectrum diseases in Europe or the US. The treatment of FTLD clinical syndromes is mostly symptomatic and the use of off-label medications is common. These off-label therapies are mostly targeted to neurotransmitter systems to normalize the brain function and unwanted behaviour, but do not target the underlying pathogenic mechanisms nor appreciably affect disease progression. Antidepressants, antipsychotics, antiepileptics, dopaminergic therapies or medications used to treat AD are commonly used to alleviate the behavioural and cognitive symptoms or motor deficits in FTLD spectrum diseases [3]. For ALS patients, riluzole and edaravone are currently the only approved drugs in Europe and the USA. However, they only increase the lifespan of the patients on average by three months [97, 98]. A summary of the current main pharmacological treatment options for patients within the FTLD spectrum is shown in Table 2 and these are discussed in the next chapters in more detail. Furthermore, the different drugs affecting specific neurotransmitter systems are indicated also in Fig. 1.

**Table 2:** Current main pharmacological treatment options of FTLD.

Pharmacological class	Therapeutic intervention	Drug	Effect	Evidence	References
Antidepressants	Behavioural symptoms	Citalopram Sertraline Fluoxetine Paroxetine Trazodone	May improve behavioural symptoms such as agitation and depression	Case reports and series, open label trials, randomised double-blind trial	[100–104, 132]
Antipsychotics	Behavioural symptoms	Quetiapine Risperidone Aripiprazole Olanzapine	May improve behavioural symptoms	Case reports and series, randomised double-blinded cross-over trial, follow-up study	[104–109]
Antiepileptics and lithium	Behavioural symptoms	Topiramate Valproic acid Carbamazepine	May improve behavioural symptoms	Case reports and series	[81, 102, 110, 112, 133, 134]
Acetylcholinesterase inhibitors	Cognitive symptoms	Donepezil Galantamine Rivastigmine	No improvement, may exacerbate behavioural symptoms	Open label trials, randomised double-blind placebo-controlled trials	[86, 117, 118]
NMDA antagonists	Cognitive symptoms, behavioural symptoms	Memantine	No improvement, may exacerbate cognition	Case series, open label trials, randomised double-blind placebo-controlled trials	[120–122, 135]
Dopaminergic therapies	Behavioural symptoms, psychotic symptoms	Dextroamphetamine Methylphenidate Selegiline Levodopa	Reduce agitation, may reduce psychotic and depressive symptoms	Randomised double-blinded cross-over trials, open label studies, case reports	[104, 105, 123, 124]

### Antidepressants

Because serotonin deficiency might contribute to the behavioural symptoms in bvFTD, increasing its levels in the brain might alleviate these symptoms. Thus, selective serotonin reuptake inhibitors (SSRI), which increase serotonin levels in the synaptic cleft, could correct the serotonergic imbalance and deficits in bvFTD patients. A recent report also showed that some antidepressants including SSRI mediate their actions by directly binding to the TrkB neurotrophin receptor. This facilitates the synaptic localization of TrkB and activation by its ligand, the brain-derived neurotrophic factor, which promotes neuronal plasticity [99]. Sertraline has shown beneficial effects in alleviating behavioural symptoms in an ALS-FTD patient [100]. In a randomised double-blind controlled crossover trial in 12 bvFTD patients, a 30-mg daily dose of citalopram alleviated the impulsivity and dysfunction of the cortical serotonergic network [101]. Furthermore, citalopram decreased irritability, depression and disinhibition in bvFTD patients in a six-week open-label study [101]. There are also positive reports from the use of fluoxetine and sertraline [76, 77]. Paroxetine has shown good efficacy in small-scale studies but no improvement of the behavioural symptoms of bvFTD patients has been observed in larger controlled studies [102]. Furthermore, trazodone, an atypical antidepressant with antagonism to multiple receptors, was effective in the treatment of irritability, agitation, depression and hyperorality of bvFTD patients in a randomised double-blind placebo-controlled study [103]. However, these potentially beneficial effects would need confirmation in larger randomized clinical trials.

### Antipsychotics

Evidence for treating the behavioural symptoms, especially agitation and violent behaviour, of bvFTD patients with antipsychotics is limited. Quetiapine has been shown to decrease agitation in a small-scale study [104], but a double-blinded study with eight bvFTD patients showed no improvement of cognitive nor behavioural symptoms [105]. In a 24-month follow-up study with 17 bvFTD patients, olanzapine decreased the overall behavioural symptoms, delusions and caregiver burden [106]. However, using atypical antipsychotics to treat the behavioural symptoms related to dementia is associated with a higher mortality risk [107]. Although increased mortality is associated with antipsychotics use in older AD patients, there is no or little evidence of this in bvFTD patients. Other severe adverse effects and the overall risk-benefit ratio should be carefully considered before using antipsychotics for the behavioural symptoms of bvFTD. Patients with FTLD spectrum diseases are also known to display extrapyramidal symptoms [67]. Especially patients with bvFTD may show symptoms of parkinsonism [108]. Extrapyramidal symptoms are common adverse effects of antipsychotics, to which the FTLD patients are vulnerable due to the dopaminergic network deficits [109]. Thus, the newer generation of antipsychotics, having less severe extrapyramidal adverse effects, should be preferred and the dosage should be the lowest possible.

### Antiepileptics and lithium

Antiepileptics to treat chronic pain and mood stabilisers in bipolar disorder patients have been successfully used [110]. They could be beneficial also for treating FTLD because they stabilise excess neuronal excitation [32, 43, 111]. One of these agents is retigabine, which activates K_v_7-potassium channels and reduces neuronal excitation. Retigabine has been shown to increase the survival of human iPSC-induced motor neurons carrying the C9-HRE [24]. Other antiepileptics such as valproic acid and carbamazepine have been reported to reduce behavioural symptoms. Carbamazepine decreased inappropriate sexual behaviour in one bvFTD patient [112] and valproic acid has been shown to decrease agitation [104]. Topiramate is an antiepileptic with a mood-stabilising action but its adverse effects include depression, suicidal behaviour, weight loss and anorexia [113, 114]. It is an AMPA and kainate receptor antagonist and has been used for binge eating disorder. Case reports have shown normalised drinking and eating behaviour and remarkable weight loss with topiramate treatment [113, 114]. As mentioned earlier, the AMPA receptor composition is altered in FTLD and especially bvFTD patients show abnormal eating behaviour [59]. Thus, antagonising AMPA receptor function might alleviate disease symptoms. Finally, lithium is used for the treatment of bipolar disorder and has multiple effects on neuronal function. Lithium might restore the deficits in autophagy in FTLD [115] but there is no clinical evidence for its use in FTLD spectrum diseases.

### Medications used in Alzheimer’s disease

AD is the most common neurodegenerative disorder characterised by loss of cholinergic neurons, and subsequent decline of memory and other cognitive functions [116]. Cholinesterase inhibitors rivastigmine, galantamine and donepezil, which increase acetylcholine levels in the brain, are widely used to treat AD patients. Rivastigmine has been reported to alleviate the behavioural symptoms to some extent but without an effect on cognitive abilities of bvFTD patients [117]. Galantamine has not shown any benefit in bvFTD patients but might have positive effects in aphasic PPA patients [86]. Donepezil in fact worsened the behavioural symptoms in a subset of FTLD patients [118], suggesting that these drugs may not be efficacious or suitable in general for symptomatic management of FTLD spectrum diseases. Moreover, theses medicines may not show efficacy since there are no cholinergic neurotransmitter system deficits in FTLD spectrum diseases [61].

The NMDA receptor-mediated excitotoxicity may be linked to FTLD. Memantine, an NMDA receptor antagonist, is used in the treatment of AD [119] but the results in bvFTD patients have not been promising. A six-month open-label study with memantine revealed no benefit for patients with FTLD-related syndromes [120]. A case series and one open-label study showed some benefits of memantine in relieving the behavioural symptoms of bvFTD patients [121] but a randomised placebo-controlled study indicated no clear benefit. In contrast, cognitive performance of bvFTD patients was decreased [122].

### Dopaminergic therapies

Because dysfunction of the neuronal dopaminergic network has been reported in FTLD, elevating dopamine levels in specific brain areas might relieve disease symptoms. Dextroamphetamine, an inhibitor of serotonin and dopamine transporters, together with the antipsychotic quetiapine improved the behavioural symptoms and decreased apathy in bvFTD patients compared to control subjects. However, quetiapine alone showed even better efficacy in alleviating these symptoms [105]. Treatment of bvFTD patients with methylphenidate, an inhibitor of dopamine and noradrenaline reuptake, was reported to improve decision-making behaviour in a double-blinded placebo-controlled study [123]. Selegiline, an inhibitor of monoamine oxidase B, which increases dopamine levels in the brain, has been shown to improve decision-making and reduce risk-taking behaviour in bvFTD patients [124] in small randomized, double-blinded cross-over trials. Finally, the dopaminergic drug levodopa might restore the altered dopamine levels and alleviate the behavioural and motor symptoms of bvFTD. However, conclusive evidence favouring the use of levodopa for FTLD spectrum diseases is still lacking and only patients with parkinsonism have been shown to benefit from dopaminergic pharmacological intervention [104].

## Limitations of current drugs and future directions

In summary, current therapies available for the treatment of FTLD spectrum diseases are mainly off-label therapies, aimed especially for symptomatic treatment of the behavioural disturbances associated with bvFTD. There are currently no pharmacological agents for the treatment of the speech deficits in PPA patients to complement speech therapy and counselling. Furthermore, there are no specific therapies for different genetic forms of FTLD. The efficacy of current pharmacological treatments is often unclear and limited due to the small scale and lack of randomisation in clinical studies. Moreover, the relative rarity of the FTLD spectrum diseases that limits the number of participating patients, the use of heterogeneous patient cohorts with different subtypes of FTLD, and variable clinical classification criteria might affect the study outcomes. However, the patients carrying C9-HRE, *MAPT* or *GRN* mutations can be identified by genetic testing. Thus, the genetic or clinical FTLD subtype should be taken into account when selecting patients for future clinical trials.

Even though FTLD comprises a group of neurodegenerative disorders partially affecting the same brain areas and neurotransmitter systems as AD, medications used for AD patients have shown few benefits or even have worsened the outcomes in FTLD clinical syndromes. Moreover, these studies have often been under-powered and used open-label drugs. Some SSRI have shown promising effects in small-scale randomised double-blind controlled studies, open-label studies and case reports, prompting further randomised placebo-controlled trials in clinically and genetically defined bvFTD patient groups. In addition, well-controlled studies are needed to assess the risk-benefit ratio of the initial promising effects of antiepileptics in the treatment of behavioural symptoms. The extrapyramidal adverse effects of antipsychotics can be difficult, and their pharmacokinetic interactions are common, which may complicate their potential. The age-dependent physiological changes affecting the pharmacokinetic properties and active metabolite formation of these drugs might contribute to the treatment efficacy, but so far these have not been studied in FTLD spectrum diseases [125].

In addition to therapeutics targeting the neurotransmitter systems, emerging therapeutic options directly targeting the fundamental molecular mechanisms of the disease such as those related to the C9-HRE or other genetic alterations by antisense oligonucleotides, micro-RNAs or small molecules have been reported [126–129]. These have been discussed in several recent reviews and will therefore not be considered here. However, an approach directly targeting the molecular mechanisms of the genetic forms of FTLD would be a major breakthrough if the treatment could be started early enough, e.g., in presymptomatic patients. Moreover, such disease-modifying therapies in combination with current symptomatic treatments that target specific neurotransmitter systems, might show benefits later during the disease. On the other hand, specific molecular mechanisms underpinning the disease may be more difficult to identify in patients who do not carry any known disease-causing genetic mutations. All in all, on the way to potential personalised treatment options at different phases of the disease, further studies are needed to verify the safety and the therapeutic outcomes of the current and future therapies in FTLD with different genetic backgrounds and clinical manifestations.
